# From Panels to Pathogen Networks: The Expanding Role of Targeted Sequencing in Veterinary Medicine

**DOI:** 10.3390/biology14081075

**Published:** 2025-08-18

**Authors:** Jiali Luo, Wentao Lu, Ruiting Liu, Shukai Zhang, Jie Cao, Chong Ma

**Affiliations:** College of Veterinary Medicine, China Agricultural University, Beijing 100193, China; sy20243051213@cau.edu.cn (J.L.); sy20233051130@cau.edu.cn (W.L.); sy20243051229@cau.edu.cn (R.L.); 2020305010419@cau.edu.cn (S.Z.)

**Keywords:** targeted sequencing, animal pathogen, enrichment strategy, one health

## Abstract

Targeted sequencing, a pivotal branch of next-generation sequencing (NGS), enables the selective enrichment of specific genomic regions and has demonstrated significant advantages in the detection of animal pathogens. This review systematically explores the underlying principles of targeted sequencing, various enrichment strategies—including PCR amplification, probe hybridization, and CRISPR-Cas systems—and their key applications in veterinary pathogen diagnostics. Due to its high throughput, sensitivity, and cost-effectiveness, targeted sequencing has been successfully applied in the multiplex detection of pathogens in economically significant livestock, such as cattle, as well as in the surveillance of antimicrobial resistance (AMR) genes, pathogen typing, and source tracing. It is particularly effective in identifying mixed infections and low-abundance pathogens. Nonetheless, wide application is restricted by some factors, like incomprehensive reference databases, cost-effectiveness, and limited application in primary-level laboratories. Further development directions are AI-based panel design, multimodal diagnostic platform integration, standard workflow construction, and introduction of a multi-omics method. Such progress focuses on enhancing the targeted sequencing scalability and precision consistent with the “One Health” initiative objective.

## Simple Summary

1. Summarize targeted sequencing enrichment strategies, including CRISPR-based depletion and capture approaches. 2. Highlights key applications in veterinary diagnostics: multipathing detection, AMR surveillance, and source tracing. 3. Comparison of targeted sequencing performance across host species via real-world case studies and data. 4. Current limitations, such as incomplete pathogen databases and a lack of standard analytical pipelines, should be identified. 5. Future directions: AI-assisted panel design, portable diagnostics, and integration with multiomics tools.

## 1. Introduction

Targeted sequencing has been developed as a high-throughput detection approach based on next-generation sequencing (NGS). It possesses the major merit of specifically enriching target sequences by means of hybridization or amplification, which can thus help precisely analyze defined genomic regions [1,2,3]. It is widely used in different fields, like oncogene identification, genetic disease screening, viral variant monitoring, and antimicrobial resistance (AMR) surveillance. Moreover, it has become a candidate for veterinary diagnosis [4,5].

Because of the high throughput, sensitivity, and cost-effectiveness [6], this technology is promising in large-scale animal pathogen detection and epidemiological surveillance. As animal pathogens are diverse and transmit rapidly, they may cause substantial economic losses to the livestock industry. Additionally, zoonotic pathogens can severely threaten public health. Therefore, it is necessary to develop diagnostic methods with high speed, accuracy, and efficiency in veterinary practice to control infectious diseases and manage animal health.

Accurate and timely diagnosis is essential for effectively controlling the occurrence and spread of diseases in veterinary medicine. With ongoing technological advancements, diagnostic approaches have become increasingly diverse. The development of isothermal amplification techniques has enabled the implementation of point-of-care testing (POCT), which is particularly well-suited for rapid screening during outbreaks in resource-limited settings, such as livestock farms [7,8]. In the clinical diagnosis of companion animals, disease-specific biomarkers allow for more precise identification of conditions, facilitating early detection and timely treatment [9]. Moreover, accurate veterinary diagnostics play a critical role in controlling the transmission of zoonotic diseases, thereby contributing to public health safety [10,11,12]. As a next-generation molecular diagnostic technology, next-generation sequencing (NGS) offers broad applications, ranging from genetic disease screening to pathogen detection. NGS enables targeted enrichment of samples to enhance the detection of specific organisms, thereby addressing the limited sensitivity or inability of conventional methods to identify certain microbes. Although NGS has not yet supplanted traditional diagnostic techniques, it remains one of the most powerful tools currently available [13,14].

Traditional diagnostic approaches, such as microbial culture, PCR, and serological assays, are extensively used, but they display the disadvantages of low throughput, decreased reliability when there are diverse pathogens, and postponed turnaround times [6]. On the contrary, targeted sequencing helps simultaneously detect several known pathogens in an individual assay, thus improving diagnostic breadth and precision. Moreover, it demonstrates the obvious advantage in identifying AMR genes and detecting complex coinfections [15,16].

The present work reviews the latest progress in applying targeted sequencing in veterinary medicine, especially its merits in detecting animal pathogens. It illustrates promising developments, like the optimization of detection panels, the advancement of automated workflows, and the integration with complementary methodologies such as metagenomic sequencing, all aimed at improving the accuracy and efficiency of veterinary pathogen diagnostics.

## 2. Principles of Targeted Sequencing and Target Enrichment Strategies

### 2.1. Technological Development and Core Characteristics of Targeted Sequencing

**Figure 1:**
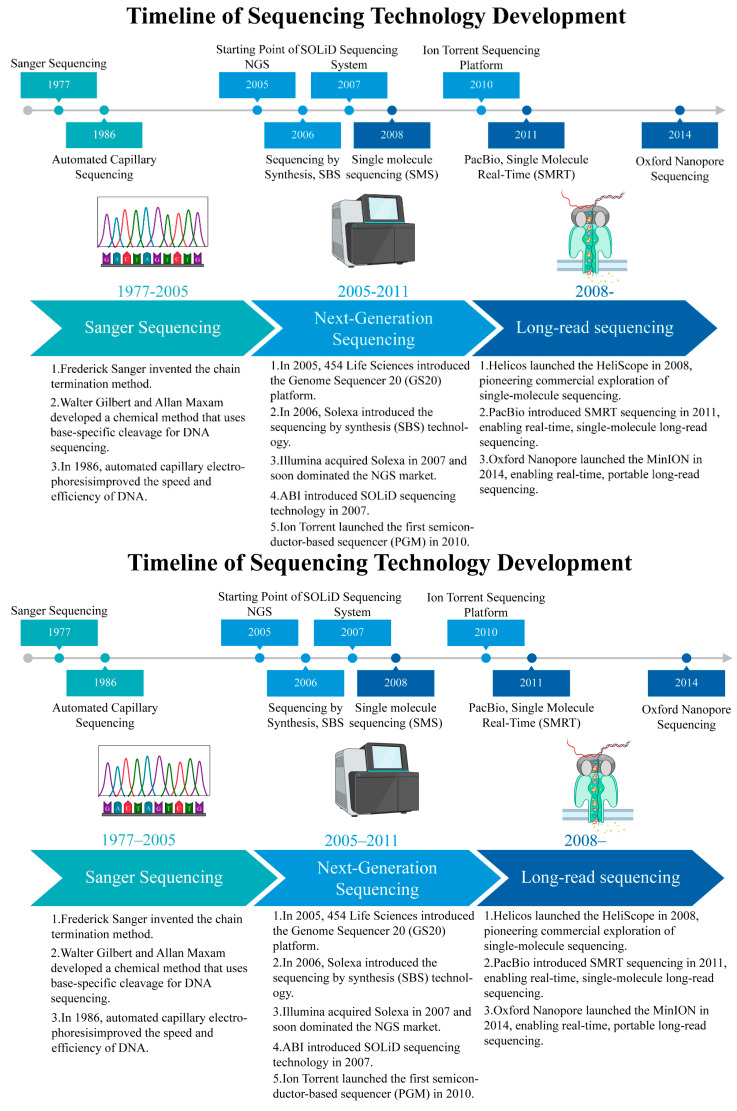
Timeline of sequencing technology development.

Sequencing technology has substantially developed, which evolved from the first-generation Sanger sequencing that was highly accurate but had low throughput in 1977 to second-generation high-throughput sequencing (NGS) in around 2005. NGS allows for massive parallel sequencing, remarkably decreases costs, and is widely applied in clinical diagnostics and genomics. In 2011, third-generation single-molecule sequencing (TGS) emerged, involving platforms like PacBio and Oxford Nanopore, and it offered markedly extended read lengths and enhanced complex genome resolution [17,18,19] (Figure 1).

Recently, the world has witnessed the evolution of Oxford Nanopore technology (from 2015 onward), which incorporates real-time and single-cell nanopore sequencing, and thus promotes progress in individualized treatment and precision medicine [20]. In the future, it may emphasize extended read lengths, decreased costs, multiomics integration, and improved sequencing accuracy, thus promoting its wider use in genetic research and clinical practice [21].

As a high-throughput sequencing approach, targeted sequencing focuses on interesting genomic regions [22]. It allows for the selective capture of predefined genes or genomic segments—ranging from dozens to thousands—which often include disease-associated gene panels or known mutational hotspots [23]. Restricting the sequencing scope produces markedly less data, which can thus simplify downstream bioinformatics analyses and enable deep targeted region coverage. Such great depth substantially improves the sensitivity in low-frequency mutation detection [24,25]. Additionally, this greater coverage enables it to identify small genomic lesions and facilitates early disease screening [26].

Targeted sequencing is associated with merits like fast turnaround time and cost-effectiveness; therefore, it is suitable for detecting known genetic variants. On the contrary, whole-genome sequencing (WGS) and whole-exome sequencing (WES) can be used to discover new variants. Nonetheless, due to the restriction of targeted sequencing to predefined genomic regions, it is incapable of detecting mutations outside the target panel. Consequently, WGS and WES are necessary for identifying new pathogenic genes under undiagnosed conditions [27,28,29,30]. Thanks to the above features, targeted sequencing is especially suitable for high-throughput diagnostics and massive sample screening.

### 2.2. Target Enrichment Strategies for Targeted Sequencing

**Figure 2:**
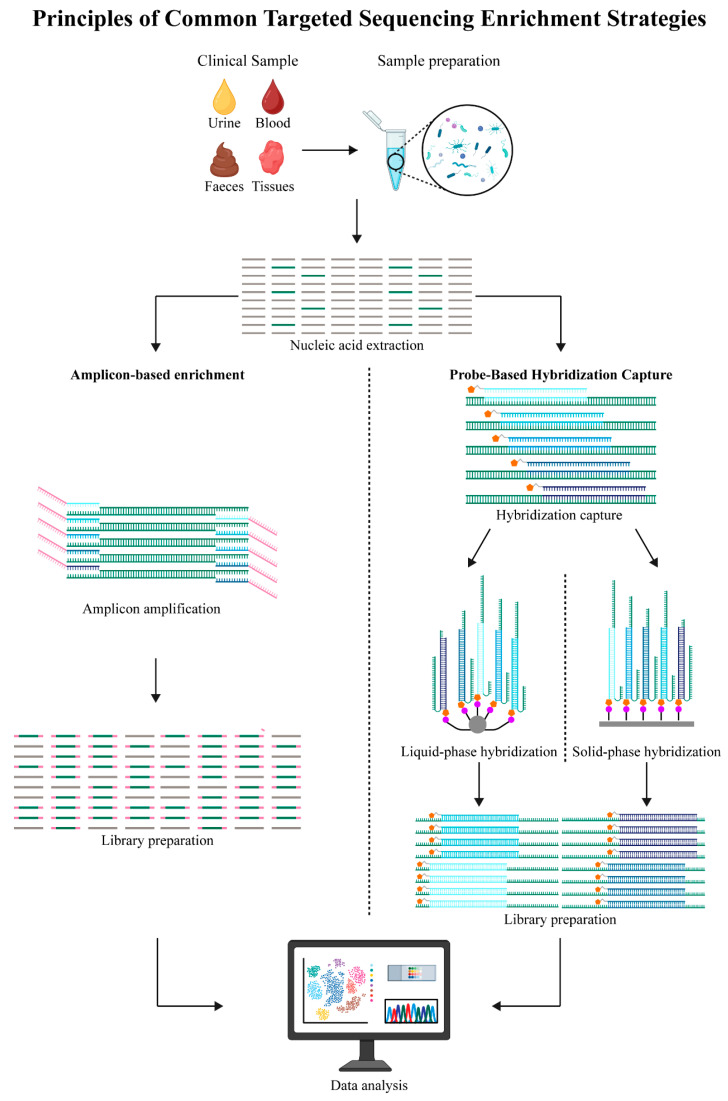
Principles of common tagged sequencing (TS) enrichment strategies. Amplicon-based enrichment strategies (**left**), probe-based hybridization capture (**right**).

**Amplicon-based enrichment:** Amplicon-based enrichment, called PCR-based enrichment as well, leverages PCR in the selective amplification of target DNA regions to increase the sequencing depth for certain genomic loci in NGS applications [2] (Figure 2). Its fundamental principle is to use sequence-specific primers for amplifying the defined genomic regions of interest, thereby effectively narrowing the sequencing scope, enhancing detection sensitivity, and enabling compatibility with low-input DNA samples such as cell-free DNA (cfDNA) and formalin-fixed, paraffin-embedded (FFPE) tissues [31,32,33].

The above method evolves from single-gene amplification (PCR) to multiplex PCR, allowing the simultaneous amplification of several target loci. However, traditional multiplex PCR may be restricted by problems like primer dimer generation, heterogeneous amplification efficiency, and competitive amplification effects [34,35,36]. High-throughput multiplex PCR techniques, like ultrahigh-multiplex or microfluidic PCR, have greatly improved detection throughput and uniform amplification; as a result, PCR-based enrichment is appropriate for massive gene panel screening [37,38,39].

Moreover, two-step PCR [40], droplet-based PCR [41], and single-molecule PCR are associated with higher amplification efficiency, higher sensitivity in low-abundance variant detection, and lower background noise. Regardless of the above progress, there are still problems related to the complex procedure and costs. At present, PCR-based enrichment is extensively used in cancer panel diagnostics, precision medicine, and AMR detection [42].

**Probe-Based Hybridization Capture:** Probe-based hybridization capture uses the longer, sequence-specific, biotin-labeled RNA or DNA probes prepared for hybridization with target DNA fragments. Following hybridization, biotin-labeled probe–target complexes can be isolated through their affinity to solid-phase supports, such as microarrays or magnetic beads, thereby facilitating the enrichment of the desired sequences [43,44,45,46].

Hybridization capture is advantageous relative to amplicon-based enrichment. It allows the targeting of more genomic regions and the design of unlimited probes, which facilitates the simultaneous capture of target sequences. Additionally, it is highly specific and has decreased amplification bias, as well as higher sequencing uniformity [47,48].

Nevertheless, probe hybridization capture is associated with some disadvantages, such as extended experimental timelines, higher costs, and greater needs for nucleic acid inputs, which have restricted its application, especially for low-yield or rare samples [22,49,50].

**Emerging Technologies for Targeted Enrichment:** As sequencing technologies develop, targeted enrichment technologies also progress quickly. Of them, the CRISPR–Cas system is the most promising, and it results in substantial targeted enrichment. Utilizing sgRNAs directs Cas proteins to specific DNA sequences to achieve selective capture. As sgRNAs are highly specific and programmable, the CRISPR–Cas system is highly flexible and can be used to enrich defined genomic regions.

**Figure 3:**
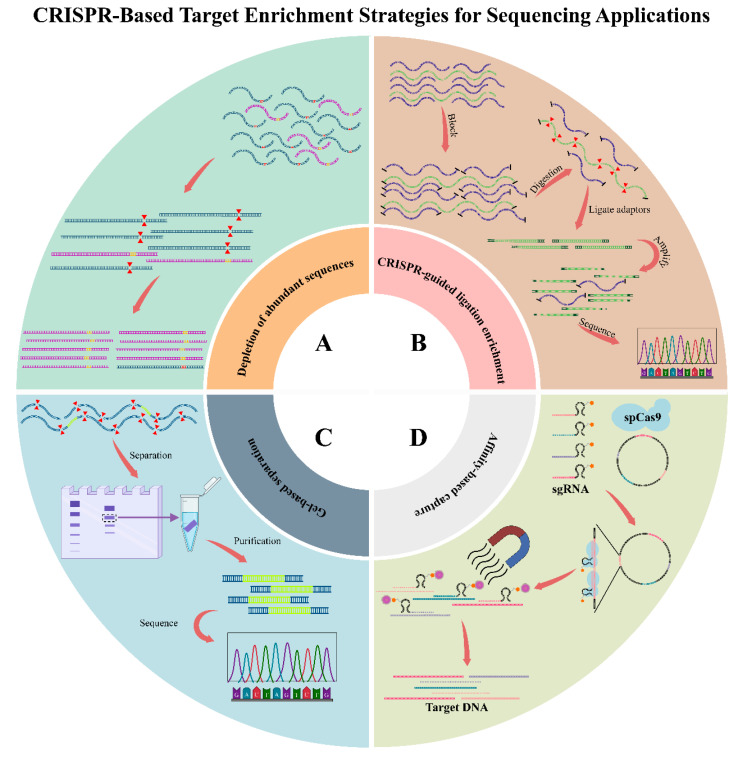
CRISPR-based target enrichment strategies for sequencing applications. (**A**) Depletion of abundant sequences. (**B**) CRISPR-guided ligation enrichment. (**C**) Gel-based separation. (**D**) Affinity-based capture.

At present, multiple CRISPR–Cas-based enrichment methods are used, such as the following:

1. Abundant sequence depletion: in the CRISPR–Cas9 system, its protospacer adjacent motif (PAM) sequence is specific, which is utilized for differentiating non-target DNA sequences; as a result, Cas protein is capable of the selective degradation of such sequences and the enrichment of target sequence. The strategy is utilized in DASH (Depletion of Abundant Sequences by Hybridization) [51] and CUT-PCR (CRISPR-mediated Ultrasensitive Detection of Target DNA via PCR) [52], efficiently removing high-abundance background sequences for improving the sensitive and specific downstream analyses (Figure 3A) [53,54].
2. CRISPR-guided ligation enrichment: Different from depletion-based technologies, the strategy cleaves target sequences under the mediation of Cas9 and the guidance of sgRNAs. After cleavage, the selective ligation of DNA fragments with adaptors is completed, which enables preferential amplification and later sequencing, whereas the uncleaved non-target DNA is not ligated and thereby eliminated from analysis. Typical technologies utilizing the as-mentioned mechanism are FLASH (Finding Low Abundance Sequences by Hybridization) [55] and FUDGE (FUsion Detection from Gene Enrichment) [56]. These two technologies utilize CRISPR–Cas-mediated enrichment for selectively capturing certain genomic regions to conduct deep sequencing analyses (Figure 3B) [54,57,58].
3. Gel-based separation: Target DNA fragments are physically separated through gel electrophoresis after CRISPR-mediated cleavage based on size differences. It is used in applications like CRISPR-mediated isolation of specific megabase-sized regions (CISMR) [59] and Cas9-assisted targeting of chromosome segments (Cas9-assisted targeting of chromosome segments, CATCH) [60], which enable the enrichment and downstream analysis of large, specific genomic regions (Figure 3C) [61].
4. Affinity-based capture: sgRNAs and modified Cas proteins can be utilized for the specific binding of target DNA sequences, and the latter undergo purification with magnetic beads. It is used in CRISPR–Cap [62] that can efficiently and specifically enrich target regions with no need for ligation or cleavage (Figure 3D) [53,63,64,65].

Since CRISPR-based enrichment is highly specific and programmable, it can be used in targeted sequencing. Nonetheless, some limitations have restricted its wide application, like the dependence on PAM sequences, difficulties in designing sgRNAs, unstable Cas enzyme activity, and lower throughput. Therefore, it is necessary to improve the performance and versatility of this method. Currently, CRISPR-based enrichment is mainly used as a complementary strategy, which does not substitute traditional enrichment strategies like amplicon-based or hybridization-based approaches.

## 3. Applications of Targeted Sequencing in the Detection of Animal Pathogens

**Multi-pathogen Detection:** As molecular diagnostic techniques develop rapidly, targeted sequencing has been increasingly identified as a promising approach in epidemiological studies and veterinary pathogen diagnostics since it is highly sensitive, specific, and has high throughput. For veterinary clinical practice, which is featured by the high mixed infection frequency and great livestock populations, comprehensive screening is necessary for determining the existing pathogen spectrum. Targeted sequencing can efficiently solve this problem by simultaneously detecting several pathogens in one assay.

For example, Lee et al. used targeted sequencing to detect pathogens in domestic cats [66], cougars, and bobcats. Anis et al. proposed a targeted sequencing method in 2018 [67], which encompassed 43 cattle and small ruminant-related pathogens, and later established a panel to detect 62 specific pathogens. The above approaches have significantly improved sensitivity and specificity, suggesting that targeted sequencing can be used in detecting multiple pathogens and species. Therefore, it may be utilized in herd-level disease monitoring and large-scale pathogen screening (Table 1).

**AMR Gene Surveillance:** In the One Health framework, the health of human beings, animals, and the environment is closely associated. AMR gene surveillance in veterinary pathogens is necessary for preventing and controlling animal diseases. Additionally, it may greatly affect public health and environmental safety [72]. AMR genes usually have a low frequency during metagenomic sequencing, so they can hardly be detected. In these situations, targeted sequencing can help enrich certain resistance-associated sequences, reduce sequencing costs, and enhance surveillance efficiency.

In addition, the prevalence of AMR is significantly higher in developing countries than in developed nations, underscoring the urgent need for AMR gene monitoring at all levels to better understand its dynamics in these regions [73]. Veterinarians, therefore, play a pivotal role in AMR surveillance. While targeted NGS does not provide full-length genome information, it demonstrates strong capability in detecting specific resistance genes and can be effectively utilized for monitoring particular pathogens [74,75]. These advantages make targeted NGS particularly suitable for the rapid establishment of veterinary AMR surveillance systems in resource-limited settings [76].

Targeted sequencing is extensively utilized in human medicine to monitor AMR during bacterial infections [77,78,79,80,81]. In veterinary environmental and clinical studies, the multiplex targeted enrichment method proposed by Yiming Li et al. shows comparatively high performance, which enables effective assessment of resistome transmission risks in complex sample matrices [82].

Regardless of the above progress, AMR gene monitoring is less developed in veterinary medicine than in human healthcare. The void is probably associated with some disadvantages, like costly sequencing and equipment, susceptible clinical samples to environmental contamination, diverse host species, and non-strict antibiotic use regulation in veterinary practice. Collectively, the above factors restrict the establishment of systematic AMR surveillance frameworks in veterinary medicine.

**Pathogen Mutation Typing and Source Tracing:** Targeted sequencing is the main high-throughput sequencing branch, which can precisely enrich genomic regions and may be potentially used in animal pathogen studies. Sequence-specific probes and primers can be used to capture genome segments or pathogenic genes, thus enabling sensitive and specific detection and reducing costs. In this regard, it is extremely efficient in the identification and characterization of less abundant pathogens.

Due to the increasing diversity of sample sources, targeted sequencing has become more and more adaptable; therefore, it enables cross-sector monitoring encompassing environmental, intermediate host, and host samples. Itarte et al. used targeted capture sequencing to detect a range of human and swine viruses at pig farms and wastewater treatment facilities [83]. They delineated the possible transmission routes based on viral genotyping data, which offer molecular evidence for assessing occupational risks and environmental monitoring [83]. In a study conducted in the Middle East and South Asia, targeted sequencing was utilized to characterize Mycoplasma gallisepticum in poultry, revealing that migratory birds may act as potential transmission vectors and enhancing our understanding of interregional transmission pathways of avian pathogens [84]. Furthermore, Megan et al. carried out a tNGS-based investigation in South Africa, demonstrating that the transmission of Mycobacterium bovis across livestock–wildlife–human interfaces in the KwaZulu-Natal Province was primarily driven by shared water sources. This study provides a scientific framework for managing zoonotic diseases in underdeveloped regions [85]. Likewise, researchers used the complex capture system for detecting PRRSV (Porcine Reproductive and Respiratory Syndrome Virus) and FLUAV (Influenza A Virus) transmission in pig farms, and confirmed phylogenetic relations across strains and underscored the usefulness of targeted sequencing in monitoring multiple hosts and environments [86].

Targeted sequencing is increasingly used in arthropod-borne pathogen monitoring. For instance, Osikowicz prepared the MPAS pipeline, which enabled high-throughput, flexible, and high-resolution tick-borne pathogen analyses [87,88] (https://doi.org/10.1186/s13071-025-06919-4).

Moreover, targeted sequencing becomes an important approach to reconstruct cross-species transmission networks in wildlife studies. Scientists targeted the RdRp gene and performed phylogenetic analysis to detect several gamma and delta coronaviruses from wild birds, revealing the tight genetic relations with zoonotic strains and suggesting the feasibility of wild waterfowl as viral reservoirs [89]. Consistently, researchers conducted deep amplicon sequencing to detect widespread human-infective Echinococcus multilocularis haplotypes from coyotes and foxes, and highlighted that wildlife was important for maintaining natural transmission chains [90]. The above results suggest that targeted sequencing is feasible for monitoring pathogens in the field.

Also, targeted sequencing is advantageous in mixed-infection and genotyping analyses. Deep amplicon sequencing targeting the PRRSV ORF7 gene was conducted to illustrate coinfection by wild-type and vaccine strains through SNV frequency analysis. Although only one gene was analyzed, barcoding was combined with parallel sequencing to implement population-level lineage monitoring [91]. Michelet et al. (2023) traced transmission networks, as well as spatial–temporal evolution in several French regions by using the 88-site single-nucleotide polymorphism (SNP) panel of Mycobacterium bovis, and demonstrated that this approach was suitable for analyzing the structure of regional pathogen population [92].

Progresses in TGS (like Nanopore) expand targeted sequencing to full-genome applications. King et al. (2021) prepared the tiled primer panel that covered the intact bovine viral diarrhea virus genome and recovered high-quality viral genomes in clinical samples through nanopore sequencing, and the coverage was as high as 99% [93]. de Vries et al. (2022) used the method in the portable, field-deployable platform, amplifying the full-genome and HA subtype of avian influenza viruses in wild bird fecal samples [94]. The method is efficient and practicable in targeted amplification when used in combination with third-generation sequencing for fast outbreak response in the field [94].

Collectively, targeted sequencing, which is highly precise, scalable, and versatile, shapes the integrated framework to detect pathogens and trace sources in environmental, wildlife, and domestic interfaces. The One Health paradigm is a cornerstone technology to monitor animal diseases, map transmission paths, and monitor population-level evolution.

## 4. Advantages and Limitations of Targeted Sequencing in Detecting Animal Pathogens

Targeted sequencing, facilitated by specific probe-based enrichment strategies, offers substantial technical advantages for pathogen detection. Through the selective capture of genomic sequences from lowly abundant pathogens, the method remarkably improves sensitivity and specificity and achieves 10- to 1000-fold increased microbial nucleic acid enrichment in comparison with metagenomic next-generation sequencing (mNGS) [22,95]. Moreover, it decreases background noise compared with mNGS and WGS; therefore, it is especially suitable for precisely identifying trace pathogens from clinical samples. As this platform has a high-throughput capacity, it can simultaneously process hundreds of samples; furthermore, the adaptable enrichment strategies allow for multiplexed detection [96,97]. This substantially enhances the disease screening efficiency in livestock, including pigs, sheep, and cattle, particularly in large-scale farming operations.

Due to the customizability, targeted sequencing is flexible in research. Panels are specifically customized for targeting separate pathogens or prepared for capturing conserved genomic regions and critical resistance-related mutations [98,99], so as to simultaneously detect pathogens and profile AMR. Progresses like CRISPR-based capture substantially improve enrichment efficiency by 100- to 800-fold, which facilitates the detection of uncommon targets from clinical samples [63,100].

In complex matrices like host-derived (feces or tissues) and environmental (like water or soil) mixed samples, targeted sequencing can employ high-specificity enrichment protocols to solve the problem of low-abundant target sequences [101]. Optimal workflows suggest the multifold target enrichment and irrelevant sequence reduction [102], providing technical support for cross-compartment surveillance and source tracing in public health.

Regardless of the above strengths, targeted sequencing is restricted to its use in veterinary medicine. It is suitable for known pathogen detection based on databases like NCBI and BV-BRC [103,104]; it can not identify unknown pathogens, so mNGS is still necessary [105]. Additionally, the targeted sequencing accuracy is greatly dependent on the currency and comprehensiveness of reference databases. Currently, international databases such as NCBI and BV-BRC encompass a wide array of microbial species and host vast amounts of data, making them well-suited for pathogen information archiving. While many regional databases have been established at the national level, limitations in academic influence and citation visibility lead most researchers to prefer globally recognized platforms like NCBI, thereby impeding the growth of local databases. Moreover, existing regional genomic initiatives—such as Fiocruz’s Genomics Network (https://www.genomahcov.fiocruz.br/en/, accessed on 5 August 2025), the Asia Pathogen Genomics Initiative (APGI), and the Africa CDC Genomics Program—primarily focus on major human infectious diseases. Specialized platforms like TB-Profiler (https://tbdr.lshtm.ac.uk/, accessed on 5 August 2025), EnteroBase (https://enterobase.warwick.ac.uk/, accessed on 5 August 2025), and GISAID (https://www.gisaid.org, accessed on 5 August 2025) are limited to specific pathogens or pathogen groups. To facilitate the integration of targeted NGS (tNGS) in veterinary medicine, it is essential to develop animal pathogen-focused international and regional databases that are complementary in scope and functionality. For the time being, regional granularity and species diversity are lacking in veterinary pathogen databases, which has limited their diagnostic and surveillance efficiency. Consequently, it is important to develop regional, host- and region-specific databases in line with standard annotation protocols and strong data-sharing frameworks to broaden the use of targeted sequencing and advance the global One Health initiative.

Even though this approach is highly customized and can be used in research, it is associated with drawbacks in application in frontline veterinary environments. Many facilities are not equipped with the necessary bioinformatics resources or infrastructure to analyze large-scale sequencing data. The absence of standardized, user-friendly analysis platforms is a major bottleneck. The development of automated tools—such as one-click solutions for data quality control (QC), target alignment, resistance prediction, and visualization—would lower barriers for non-specialists. Establishing unified technical standards ensures consistency across laboratories and promotes routine adoption.

In developing regions, the implementation of tNGS remains notably constrained despite its well-documented benefits. A recent study highlighted that while countries in South and Southeast Asia are developing capabilities in pathogen genomics, these efforts are primarily focused on human health surveillance, with limited extension to environmental or veterinary contexts. Major obstacles include dependence on international funding sources, disruptions in supply chains, a shortage of skilled technical personnel, and insufficient quality control systems [106]. In response, international partnerships are increasingly being established to enhance molecular biology infrastructure in these areas and to facilitate the expanded use of NGS technologies, including tNGS, in animal disease detection and epidemiological monitoring [107,108,109].

Finally, the cost of targeted sequencing remains a key constraint, particularly compared with conventional molecular diagnostic methods such as qPCR [110].

From a procurement standpoint, qPCR instruments typically range in cost from several thousand to tens of thousands of US dollars, while NGS sequencing platforms are priced significantly higher, often between tens of thousands and several hundred thousand US dollars [111]. In commercial diagnostic kits, the cost per qPCR test generally falls within USD 30 to 100 (https://en.biovet-inc.com/wp-content/uploads/2024/12/Biovet_DOS-2025_Pricing_Bovine_EN_Digital.pdf, accessed on 5 August 2025). Recent technological improvements in qPCR have optimized cost efficiency, with some studies citing per-sample expenses as low as USD 15–30 [7,112,113].

Although comprehensive cost analyses for tNGS are relatively scarce, research by Marco et al. reports that reagent and consumable costs per sample decrease with higher throughput and sample volume. Despite these reductions, the average cost per NGS sample remains between USD 101 and USD 173, which is notably higher than for qPCR. Additionally, the overall expense of NGS includes several hidden components, such as training personnel, logistics, bioinformatics infrastructure, and quality control systems, all of which contribute substantially to total implementation costs [114].

While qPCR remains a cost-effective and rapid method for detecting specific pathogens, it is limited by its lower throughput and narrow target scope. In contrast, targeted sequencing offers greater multiplexing capacity by simultaneously detecting dozens to hundreds of pathogens and genetic markers. Research has shown that tNGS can identify pathogens overlooked by conventional diagnostics and provide advanced insights through genotyping, mutation analysis, resistance gene detection, and strain-level differentiation—capabilities that extend beyond the resolution of standard qPCR. This makes tNGS a more adaptable and information-rich option for large-scale or complex surveillance scenarios [66,67,68,69,70,71].

In cost-sensitive sectors such as animal production, diagnostic affordability, and usability are of paramount importance. To promote broader adoption, targeted sequencing should be strategically optimized by developing indication-specific panels and maximizing sample throughput, thereby reducing per-sample costs. These measures are essential to improving the economic and practical feasibility of targeted sequencing within veterinary diagnostics.

## 5. Future Development and Outlook

Sequencing technology can be likened to biological LEGO blocks—its modular nature facilitates innovative applications when integrated with diverse upstream experimental inputs. The future of targeted sequencing will extend beyond addressing current technical challenges, emphasizing its integration with artificial intelligence (AI), automated bioinformatics, and multi-omics diagnostic frameworks to accommodate the growing complexity of cross-species pathogen surveillance under the “One Health” paradigm.

**AI-Driven Panel Design:** Conventional panel design typically depends on manual selection of conserved regions in known pathogens, which can lead to limited genomic coverage and low design efficiency. The integration of AI, particularly machine learning, offers the potential to accelerate panel development through predictive modeling of primer and probe combinations, assessment of enrichment performance, and estimation of specificity. These AI-driven approaches will enable more efficient and adaptive panel generation [115]. Looking ahead, region-specific panels guided by algorithms for multi-sequence alignment and hotspot clustering may facilitate real-time adaptation to circulating pathogen strains [116].

**Integration with Multimodal Diagnostics:** A promising direction is the combination of targeted sequencing with rapid diagnostic tools such as colloidal gold assays, loop-mediated isothermal amplification (LAMP), and CRISPR-based tests [117]. This dual-mode strategy—initial field screening followed by in-depth sequencing—can increase both the speed and resolution of diagnostic methods. Moreover, deploying sequencing platforms on portable devices (e.g., MinION) may significantly increase field responses to emerging or wildlife-associated outbreaks.

**Development of Standardized, Intelligent Analytical Platforms:** To facilitate broader clinical adoption, there is an urgent need for user-friendly, automated bioinformatics platforms. “One-click” tools that integrate quality control, target alignment, resistance gene prediction, and data visualization can significantly reduce reliance on specialized personnel. The establishment of standardized protocols will ensure consistency across laboratories and geographic regions, thereby supporting routine deployment beyond research settings.

**Integration into Multiomics Pathogen Research:** Targeted sequencing is positioned to become a cornerstone of comprehensive omics-based pathogen research. When integrated with transcriptomics, metabolomics, or epigenomics, it offers the potential to yield novel insights into pathogen–host interactions. For instance, mutation-level data derived from targeted sequencing, in conjunction with metabolic profiling, may illuminate the functional consequences of resistance mutations—effectively linking genotypic variation to phenotypic expression.

**Transitioning Toward Precision, Sustainability, and Integrated Control:** Amid growing efforts to reduce antimicrobial usage and advance precision medicine, targeted sequencing is poised to become a pivotal component of data-driven surveillance and intervention strategies. When integrated with spatial epidemiological models and ecological datasets, it holds the potential to facilitate near real-time monitoring of pathogen dynamics and enable more nuanced risk assessments at regional levels.

## 6. Conclusions

As an innovative extension of next-generation sequencing, targeted sequencing is distinguished by its precision, scalability, and adaptability. It has demonstrated substantial utility in cattle pathogen surveillance through multipathogen detection, resistance gene monitoring, and genotyping. Its effectiveness is particularly pronounced in the investigation of complex infections and the identification of low-abundance targets. However, its broader adoption remains hindered by practical challenges, including incomplete pathogen databases, field-level technical limitations, and high implementation costs. To realize its full potential, future advancements should focus on dynamic AI-assisted panel optimization, integration with portable and rapid diagnostic tools, and the standardization of analytical workflows. These innovations will position targeted sequencing as a pivotal tool for livestock disease management, transboundary pathogen surveillance, and the implementation of One Health approaches. With ongoing technological advancements and interdisciplinary collaboration, targeted sequencing is set to deliver intelligent, high-efficiency solutions for livestock health, zoonotic disease preparedness, and global animal health governance.

## Figures and Tables

**Table 1:** Comparative applications of targeted sequencing in the detection of different animal pathogens.

Comparison Item	Cattle [60]	Horse [68]	Felids [66]	Cattle [69]	Feline [70]	Dog [71]
Number of Pathogens Detected	43	62	31	17	6	17
Pathogen Types	Bacteria, viruses, parasites, micromycetes	Bacteria, viruses, parasites, micromycetes	Viruses and bacteria	Bacteria, viruses, Protozoa	Bacteria, viruses	Bacteria, viruses, Protozoa
Host Species	Cattle, small ruminants	Horses	Domestic cats, bobcats, cougars	Cattle, small ruminants	Feline	
Targeted Sequencing Method	tNGS (targeted amplicon sequencing)	tNGS (targeted amplicon sequencing)	TGC-NGS (targeted genome capture sequencing)	tNGS (targeted nanopore sequencing)	tNGS (targeted amplicon sequencing)	tNGS (targeted amplicon sequencing)
Sample Types	Milk, nasal swabs, lung tissue, blood, amniotic fluid, etc.	Respiratory, reproductive, nervous, and digestive systems	Blood, serum, tissue, feces, cell culture supernatant	Placenta, amniotic fluid, vaginal swab, semen, fetal tissues	Oropharyngeal/nasal swabs, respiratory tissues	/
Multipathogen Detection	High—simultaneous detection of multiple pathogens	Very high—broader pathogen coverage	High—supports cross-species pathogen detection	High—supports cross-species pathogen detection	High	High
Low-Abundance Pathogen Detection	LOD = Ct 38	LOD = Ct 30–35	Up to 5600-fold enrichment, improves detection sensitivity	LOD = Ct 37	LOD = Ct 35–37, variability observed in SARS-CoV-2	LOD = Ct 35–36
Pathogen Typing Capability	Detects virulence, resistance, and toxin genes	Further optimized to identify resistance genes	Enables whole-genome sequencing for pathogen genotyping	Enables pathogen typing	Enables pathogen typing	Enables pathogen typing
AMR Gene Detection	Limited	Expanded AMR gene coverage	Focuses on full-genome detection of pathogens	/	/	/
Application Scope	Clinical diagnosis of infectious diseases, outbreak surveillance	Equine disease diagnostics, antimicrobial resistance monitoring	Research on felid pathogens, cross-species pathogen studies	Suitable for diagnosis and surveillance in high coinfection bovine cases	Clinical detect	Clinical detect
Bioinformatics Complexity	Requires bioinformatics analysis	Requires bioinformatics analysis	Requires advanced data processing and comparison	Open-sourced on GitHub with ready-to-use pipeline	Requires bioinformatics analysis	Requires bioinformatics analysis
Limitations	Sample type affects detection; some genes may be missed	Low-abundance detection remains challenging; high data demands	Probe design limits pathogen coverage; partial detection possible	Detection ≠ causation; interpret with clinical context	Inconsistent performance for SARS-CoV-2 detection	May miss ultra-low abundance pathogens; single time-point blood samples might fail PCR/tNGS detection
Detection Cost	High, but lower than WGS	High, but lower than WGS	Currently high (USD 450–550/sample), but may decline	Cost-effective if multiple samples are processed within a single flow cell	Cost-effective tNGS for large-scale respiratory screening	/

## Data Availability

Not applicable.

## Abbreviations

The following abbreviations are used in this manuscript:

NGS	Next-Generation Sequencing
AMR	Antimicrobial Resistance
TGS	Third-Generation Single-Molecule Sequencing
WGS	Whole-Genome Sequencing
WES	Whole-Exome Sequencing
cfDNA	Cell-Free DNA
FFPE	Formalin-Fixed, Paraffin-Embedded
sgRNAs	Single Guide RNAs
SNP	Single-Nucleotide Polymorphism
mNGS	Metagenomic Next-Generation Sequencing
LAMP	Loop Mediated Isothermal Amplification
